# Supplementary material for: LSD1 controls a nuclear checkpoint in Wnt/β-Catenin signaling to regulate muscle stem cell self-renewal
Source: Nucleic Acids Res. 2024 Feb 7;52(7):3667–81. doi: 10.1093/nar/gkae060 (PMC11040000; doi:10.1093/nar/gkae060)
Supplement: gkae060_Supplemental_File [file gkae060_supplemental_file.pdf]

LSD1 controls a nuclear checkpoint in Wnt/β-Catenin signaling to regulate muscle stem cell self-renewal

Sandrine Mouradian,[1,4] Delia Cicciarello,[1,4] Nicolas Lacoste,[1,4] Valérie Risson,[1] Francesca Berretta,[1] Fabien Le Grand,[2] Nicolas Rose,[2] Thomas Simonet,[1] Laurent Schaeffer,[1,3*] Isabella Scionti[1,*]

This file contains following material:

Supplemental Table S1. DNA/RNA oligonucleotides, antibodies, plasmids and mouse models used in this study.
Figure S1. LSD1 inactivation delays myogenic differentiation.
Figure S2. LSD1 inactivation affects early step of muscle regeneration.
Figure S3. LSD1 is required for β-catenin transcriptional activity.
Figure S4. LSD1 inactivation does not influence ESC maintenance.

Supplemental Table S1. DNA/RNA oligonucleotides, plasmids, antibodies and mouse models used in this study.

**REAGENT or RESOURCE**	**SOURCE**	**IDENTIFIER**
**<u>Experimental models: organisms/strains:</u>**		
C57BL/6J mice	Charles River	N/A
Pax7<tm2.1(cre/ERT2)Fan>/J	The Jackson Laboratories	#012476
LSD1tm1Schüle	Zhu, D et al., Nat commun 2014	N/A
**<u>Oligonucleotides:</u>**		
LSD1 Wt For genotyping: ATA-CGA-AGT-TAT-GGA-TCC-AAG	Eurogentec	N/A
LSD1 Flox For genotyping: CCT-ACA-CTG-TGC-CAG-GCT-GC	Eurogentec	N/A
LSD1 Rev genotyping: GCA-GGC-GGT-TTG-AAA-TGT-ATT-C	Eurogentec	N/A
Cre For genotyping: CGA-TGC-AAC-GAG-TGA-TGA-GG	Eurogentec	N/A
Cre Rev genotyping : GCA-TTG-CTG-TCA-CTT-GGT-CGT	Eurogentec	N/A
MyoD NEG For ChIP: CCC-TTC-ATC-CAG-GGC-ACT-AC	Eurogentec	N/A
MyoD NEG Rev ChIP: TTG-GGA-ACC-CAG-CAG-TAA-GC	Eurogentec	N/A
MyoD CER For ChIP: CTA-AAC-ACC-AGG-CAT-GAG-AGG	Eurogentec	N/A
MyoD CER Rev ChIP: ACT-CAC-TTT-CTC-CCA-GAG-TTG-C	Eurogentec	N/A
Fst For Real-Time qPCR: CTG-CTG-CTA-CTC-TGC-CAG-TT	Eurogentec	N/A
Fst Rev Real-Time qPCR: ACA-TCC-TCC-TCG-GTC-CA-TGA	Eurogentec	N/A
Axin2 For Real-Time qPCR: GGG-TTC-TGA-AAT-TCA-TAG-ACT	Eurogentec	N/A
Axin2 Rev Real-Time qPCR: CGA-CTG-TTC-AAT-AAA-TAT-CAG	Eurogentec	N/A
Ctnnb1 For Real-Time qPCR: TAC-GAG-CAC-ATC-AGG-ACA-CC	Eurogentec	N/A
Ctnnb1 Rev Real-Time qPCR: ACA-ATC-CGG-TTG-TGA-ACG-TC	Eurogentec	N/A
MyoD1 For Real-Time qPCR: AGC-ACT-ACA-GTG-GCG-ACT-CA	Eurogentec	N/A

MyoD1 Rev Real-Time qPCR: GCT-CCA-CTA-TGC-TGG-ACA-GG	Eurogentec	N/A
**Plasmids:**		
pCMX-LSD1 flag	Laboratory of Roland Schüle	N/A
pCMX-LSD1 K661A flag	Laboratory of Roland Schüle	N/A
pCMX-LSD1 K661A/W754A/Y761A flag	Laboratory of Roland Schüle	N/A
pCMV-GFP	GenScript	N/A
pCMV β-CAT	GenScript	N/A
pCMV β-CAT K180R	GenScript	N/A
M50 super 8xTOPFLASH	Addgene	#12456
M51 super 8xFOPFLASH	Addgene	#12457
pRL-TK	Promega	#E2241
shRNA against CTNNB1	Merck	TRCN0000012690
Control shRNA scrambled	Merck	SHC016H
CTNNB1 WT_mCherry_pCAGIG	GenScript	N/A
mCherry_pCAGIG	GenScript	N/A
**Recombinant proteins:**		
β-CAT WT: Biotin-GGGGGAAVMVHQLSKKEASRHAIMRSPQMVSAIVRTMQNTNDVETARCTAGTLHNLSHHREGLLAIF	Proteogenix SAS	N/A
β-CAT K180me: Biotin-GGGGGAAVMVHQLSK(me)KEASRHAIMRSPQMV SAIVRTMQNTNDVETARCTAGTLHNLSHHREGLLAIF	Proteogenix SAS	N/A
Flag: MDYKDHDGDYKDHDIDYKDDDDK	Proteogenix SAS	N/A
Recombinant LSD1	Merck	#SRP0122
**Antibodies:**		
Mouse anti-Pax7	DSHB	#Pax7
Rabbit anti-Ki67	Cell Signaling	#9129
Rabbit anti-LSD1	Abcam	#17721
Rabbit anti-Laminin	Merck	#L9393
Rat anti-MYF5	Active motif	# 39801
Mouse anti-Myogenin	DSHB	#F5D
Rabbit anti-pan methyl Lysine	Abcam	#7315
Rabbit anti-GFP	Merck	#G1544

Streptavidin-HRP	Thermo Fisher scientific	#434323
Monoclonal ANTI-FLAG® M2	Merck	#F1804
Rabbit anti- Non-phospho (Active) β-catenin	Cell Signaling	#8814
Rabbit anti-β-catenin	Cell Signaling	#9562S
Rabbit anti-H3	Cell Signaling	#4499S
Rabbit anti-BCL9	Invitrogen	#PA5-49466
Rabbit anti-Desmin	Cell Signaling	#5332
Mouse anti-Myosin	DSHB	#A4.1025
Rat anti-CD34-FITC	Thermo Fisher scientific	#11-0341-82
Rat anti-CD45-PE	Thermo Fisher scientific	#12-0451-82
Rat anti-CD31-PE	Thermo Fisher scientific	#12-0311-82
Rat anti-Ly-6A/E(Sca-1) -PE	Thermo Fisher scientific	#12-5981-82
Rat anti-Alpha 7 integrin 647	AbLab	#67-0010-05

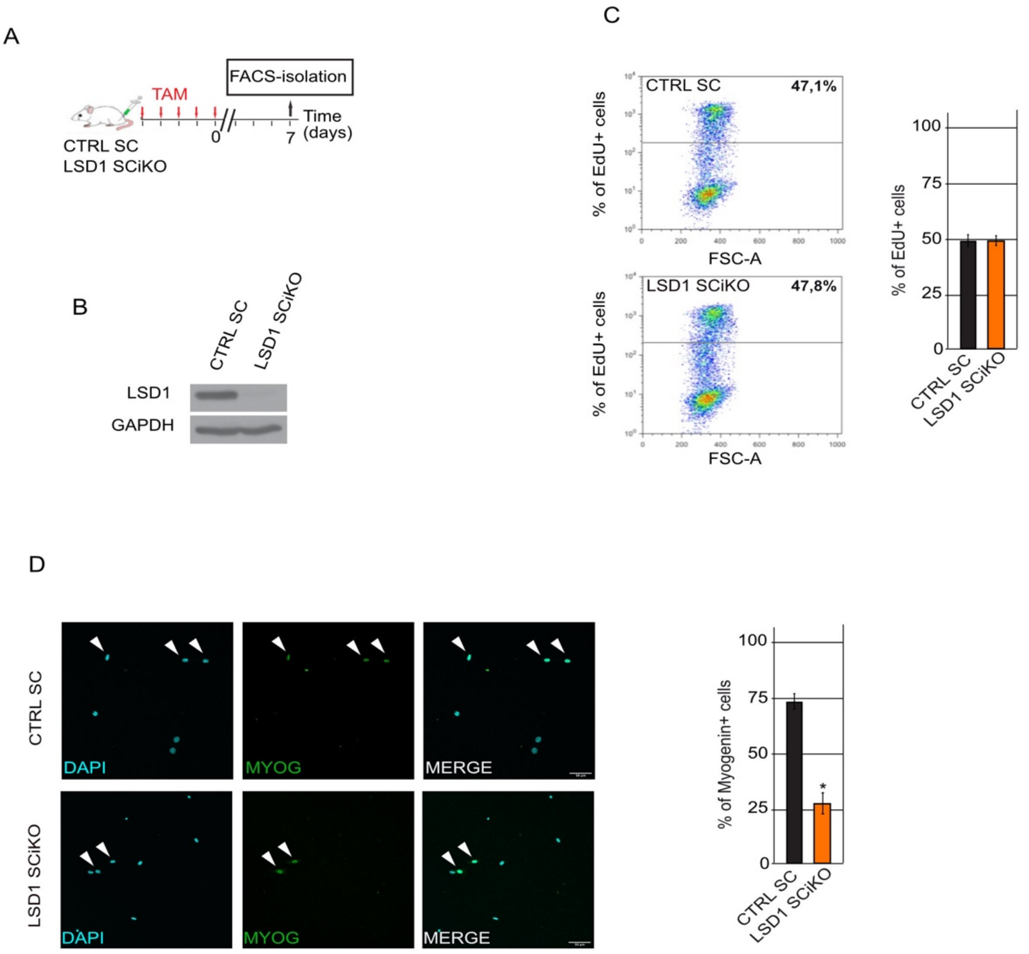

Figure S1. LSD1 inactivation delays myogenic differentiation *in vitro*. (**A**) Experimental set up. (**B**) LSD1 immunoblot on FACS-sorted MuSCs from CTRL SC and LSD1 SCiKO muscles cultured in growth medium for 4 days. GAPDH was used as loading control. (**C**) Percentage of CTRL SC and LSD1 SCiKO MuSCs in S-Phase. Measurements were made by cytometry analysis after treatment with EdU for two hours. (**D**) Myogenin immunostaining of FACS-sorted MuSCs from CTRL SC and LSD1 SCiKO muscles, seeded at low density, after 48 h of myogenic differentiation medium. Representative images of MYOG positive MuSCs are shown.
Scale bars, 50 µm. n = 5 primary cell cultures/genotype. Values are percentage mean ± SEM. *p < 0.05 (Bonferroni test after one way-ANOVA).

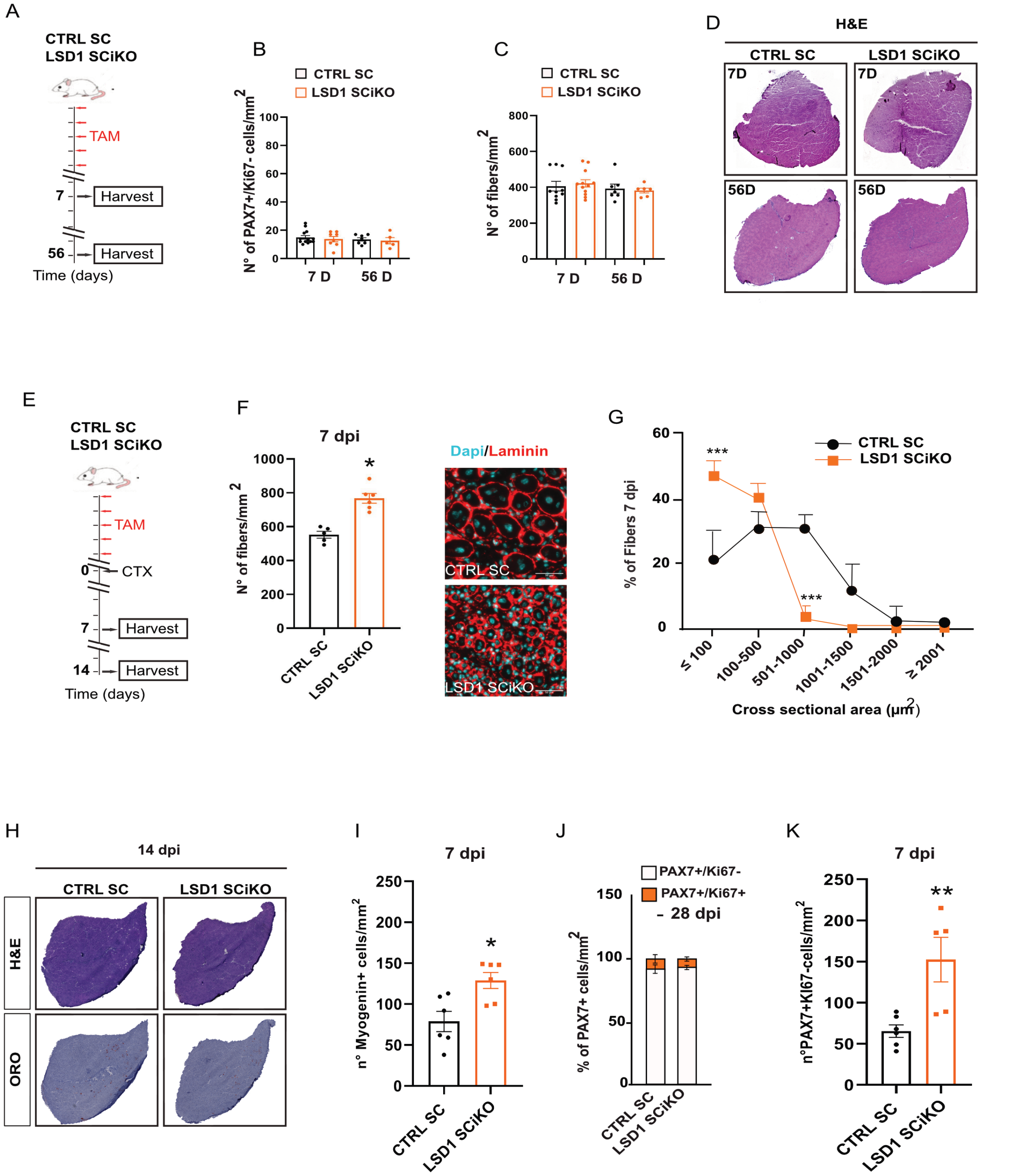

Figure S2. LSD1 inactivation affects early step of muscle regeneration. (**A**) Experimental set up. (**B**) Quantification of the number of sublaminar PAX7+/Ki67- cells per mm^2. (**C**) Quantification of the number of myofibers per mm^2. (**D**) Histological analysis of TA muscle in CTRL SC and LSD1 SCiKO mice. (**E**) CTX experimental setup. (**F**) Anti-Laminin staining on cryosections of regenerated TA muscles in CTRL SC and LSD1 SCiKO mice at 7 dpi. Quantification of the number of myofibers per mm^2. (**G**) CSA distribution of muscle fibers in CTRL SC and LSD1 SCiKO mice TA cryosections at 7 dpi. (**H**) Hematoxylin-eosin and Oil Red O staining on cryosections of regenerated TA muscles in CTRL SC and LSD1 SCiKO mice at 14 dpi. (**I**) Quantification of the number of Myogenin + cells per mm^2 in CTRL SC and LSD1 SCiKO mice at 7 dpi. (**J**) Percentage of PAX7+/Ki67+ (orange, proliferating cells)

MuSCs at 28 dpi was quantified per mm^2. (**K**) Quantification of the number of PAX7+/Ki67- cells per mm^2 in CTRL SC and LSD1 SCiKO mice at 7 dpi.
Scale bars, 50 µm. n = 4 mice/genotype. Values are mean or percentage mean ± SEM. *p < 0.05, **p < 0.006, ***p < 0.001 (Bonferroni test after one way-ANOVA).

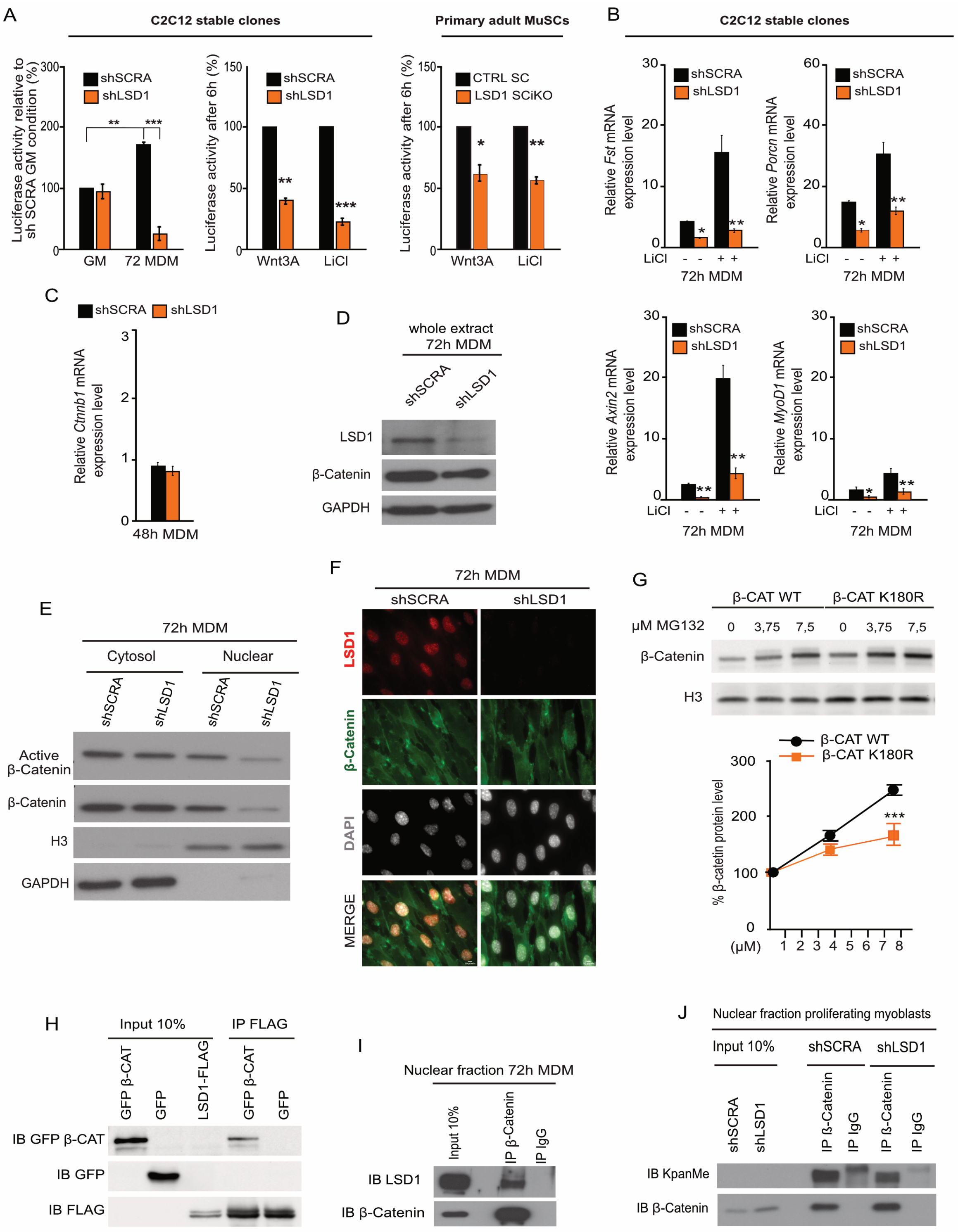

Figure S3. LSD1 is required for β-catenin transcriptional activity. (**A**) The TCF transcriptional activity of C2C12 (shSCRA and shLSD1) and MuSC (CTRL SC and LSD1 SCiKO) cells is shown as a ratio of TOP-FLASH to FOP-FLASH luciferase-mediated signals, in proliferation (GM) and after 72 hours in MDM for C2C12 stable clones and when cultured for 6 h in the presence of Wnt3A or LiCl for both C2C12 stable clones and MuSCs. (**B**) *Fst, Porcn, Axin2* and *MyoD1* mRNA levels in shSCRA and shLSD1 cells untreated and treated with LiCl during differentiation. RT-qPCR values were normalized to the *Ppib* mRNA. mRNA levels are shown as the fold

variation compared to shSCRA cells at MDM0. (**C**) *Ctnnb1* mRNA levels in shSCRA and shLSD1 cells after 48 h in MDM. RT-qPCR values were normalized to the *Ppib* mRNA. mRNA levels are shown as the fold variation compared to shSCRA cells at MDM0. (**D**) Western blot analysis of β-catenin in whole protein extract of shSCRA and shLSD1 cells after 72 h in MDM. GAPDH was used as loading control. (**E**) Western-blot analysis of β-catenin in the two cellular compartments (Cytosol: GAPDH) and Nucleus (H3). (**F**) Anti-LSD1 and anti- β-catenin immunostaining of shSCRA and shLSD1 cells after 72h in MDM. (**G**) Detection of β-catenin WT or mutant β-catenin K180R protein level in HEK 393T cells without or with treatment with MG132. (**H**) CoIP detection of the binding of LSD1 to β-catenin in transfected HEK 293T cells. (**I**) Endogenous β-catenin and LSD1 interacted with each other in the nuclear fraction of C2C12 cells after 72 h in MDM. (**J**) LSD1 does not affect the β-catenin protein methylation status in proliferating myoblasts.
Values are mean or percentage mean of at least three experiments. ± SEM. *$p < 0.05$, **$p < 0.01$, ***$p < 0.001$ (Bonferroni test after one way-ANOVA).

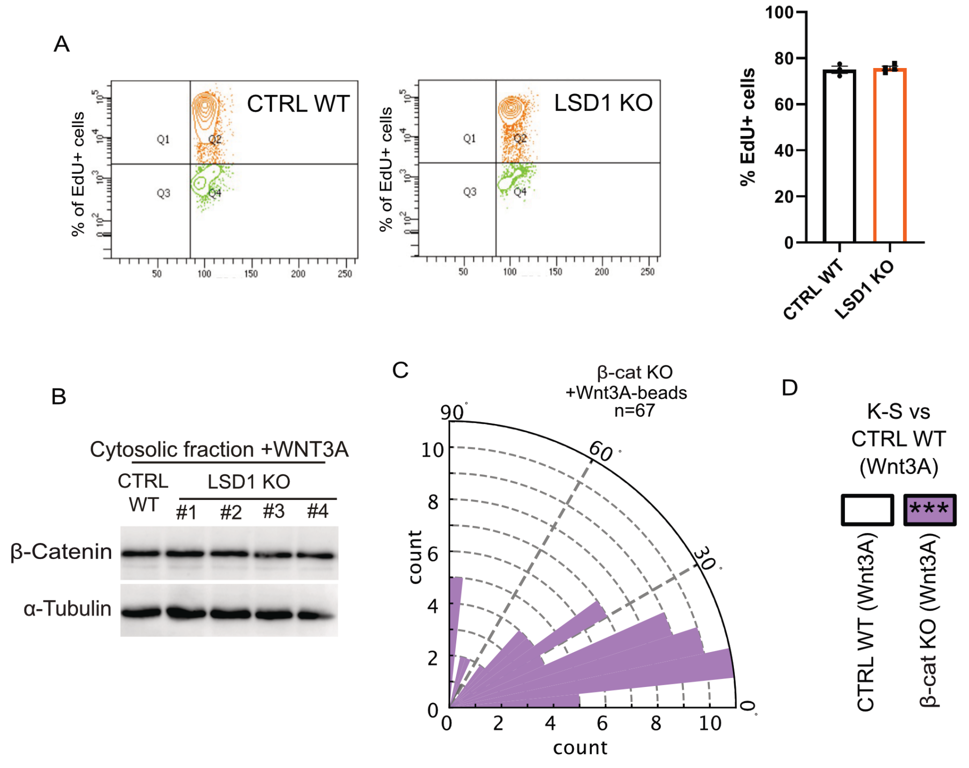

Figure S4. LSD1 inactivation does not influence ESC maintenance. (**A**) Percentage of CTRL WT and LSD1 KO ESCs in S-Phase. Measurements were made by cytometry analysis after treatment with EdU for two hours. (**B**) Western blot analysis of cytosolic β-catenin protein in CTRL WT and 4 different LSD1 KO ESCs clones. (**C**) Rose plot depicting the distribution of mitotic spindle angle orientations in β-catenin KO ESCs. n= number of cells. (**D**) ***p<0.001 in box indicates statistical significance calculated by multiple Kolmogorov-Smirnov tests against CTRL WT ESC dividing with a Wnt3a-bead.
